# Evaluation for Fasting and 2-hour Glucose and HbA_1_c for Diagnosing Diabetes Based on Prevalence of Retinopathy in a Chinese Population

**DOI:** 10.1371/journal.pone.0040610

**Published:** 2012-07-12

**Authors:** Zhong Xin, Ming-Xia Yuan, Hong-Xing Li, Lin Hua, Jian-Ping Feng, Jing Shi, Xiao-Rong Zhu, Xi Cao, Jin-Kui Yang

**Affiliations:** 1 Department of Endocrinology, Beijing Tongren Hospital, Capital Medical University, Beijing, China; 2 Department of Mathematics, School of Biomedical Engineering, Capital Medical University, Beijing, China; Johns Hopkins Bloomberg School of Public Health, United States of America

## Abstract

**Background:**

The glycemic thresholds for diabetes diagnosis have long been at the forefront of discussion. However, no information about glycemic cutoff points has been made available for the Chinese population. The aim of the present study was to examine the association of fasting plasma glucose (FPG), 2-h plasma glucose (2-h PG) and HbA_1_c levels with diabetic retinopathy (DR) and determine the associated cutoff levels in a Chinese population.

**Methodology and Principal Findings:**

In a cross-sectional population-based sample of 2551 Chinese (representing a population of 1,660,500 in a Beijing district) between 18–79 years of age, the three glycemic measures were measured in a 75 g oral glucose tolerance test, and DR was assessed by two 45° color digital retinal images. The prevalence of DR increased in the ninth decile of each variable, corresponding to an FPG of ≥7.2 mmol/l, a 2-h PG of ≥10.7 mmol/l, and HbA_1_c of ≥6.4%, according to the Joinpoint regression method. After excluding individuals receiving antihyperglycemic medication, the prevalence significantly increased at an FPG of ≥6.8 mmol/l, a 2-h PG of ≥12.0 mmol/l, and HbA_1_c of ≥6.7%. The area under the ROC curve for all three measures showed no significant differences for detecting DR. After excluding individuals receiving antihyperglycemic medication, the three measures also showed no significant differences.

**Conclusions and Significance:**

A significant increase in retinopathy prevalence occurs among individuals with FPG ≥7.2 mmol/l, 2-h PG ≥10.5 mmol/and HbA_1_c ≥6.4%; and measuring FPG or HbA_1_c are equally reliable methods as measuring 2-h PG for the diagnosis of diabetes in the Chinese population.

## Introduction

The glycemic thresholds for diabetes diagnosis have long been at the forefront of discussion. To determine such thresholds, glycemic tests calibrated against diabetic retinopathy (DR) observations have become the preferred diagnostic criterion for diabetes [1]. DR is an early and specific complication that correlates strongly with the onset of diabetes [2]. The current diagnostic cutoff values for diabetes (fasting plasma glucose [FPG] of 7.0 mmol/l and 2-h post oral glucose load plasma glucose [2-h PG] of 11.1 mmol/l) were determined from glycemic levels that associated with a significantly increased risk of DR [3], [4]. These diagnostic cutoff points were calibrated with data from several population-based studies, including those of Egyptians [1], Pima Indians [5] and the Third National Health and Nutrition Examination Survey (NHANES III) participants [3].

Various studies have found significant differences in sensitivity and tolerability to glucose loading between ethnic groups [6], [7], [8]. The glycemic cutoff points associated with DR may also be affected by the level of understanding and disease control in the sample population. The cost of treating diabetes and its complications is a major concern in China, where the prevalence of diabetes is high and a large proportion of people with diabetes remain undetected and uncontrolled [9], [10].

To date, no information about glycemic cutoff points has been made available for the Chinese population. Our objectives of this study were 1) to examine the relationships between DR prevalence and FPG, 2-h PG and HbA_1_c levels in the Chinese population; and 2) to compare the performance of the above three glycemic measures for diagnosing diabetes using the presence of DR as the true diabetes state (gold standard).

## Materials and Methods

### Ethics statement

The study was conducted with the approval from the Ethics Committee of Beijing Tongren Hospital, Capital Medical University. Written informed consent was obtained from each participant.

### Study population

Between July 2010 and March 2011, the Health Examination Survey in Beijing, a cross-sectional, population-based survey on chronic diseases and risk factors was conducted in Changping, one of the newly developing districts in Beijing with an area of 1,343.5 square kilometers and a permanent resident population of 1,660,500. Household sampling was performed by the Center for Disease Control and Prevention (CDC) of Beijing; 8,155 randomly selected households were eligible (occupants were of Chinese ethnicity and had resided in Changping for more than 6 months). All household residents 18–79 years of age were enumerated in each sampled household; then, using Kish's selection tables [1], one person was randomly selected to participate in the study (regardless of whether the person was at home during the field visit; and regardless of whether they had diabetes). Of the 8,155 individuals, 8084 received baseline examinations including a physical examination, FPG measurements, and renal and liver function tests in addition to completing a general health questionnaire. Then, 3760 subjects whose FPG ≥5.6 mmol/l were invited to perform a 75 g oral glucose tolerance test (OGTT) and ophthalmic examination by Beijing Diabetes Prevention and Treatment Office and field workers. Of a total of 3760 residents in that group, 2592 subjects (68.9%) consented to participate in the study. After excluding 31 subjects who had cataracts, 6 subjects who had glaucoma and a further 4 subjects with other eye diseases, a total of 2551 individuals successfully completed the OGTT and ophthalmic examination.

### Laboratory measurements

Blood samples were collected after an overnight fast for the determination of plasma glucose and HbA_1_c levels. After the fasting blood specimen had been taken, the OGTT was performed between 08:00 and 10:00 hours. At 120 min, a blood sample was obtained for the determination of post-loading plasma glucose levels. These specimens were analyzed within 24 h. Plasma glucose was determined by the glucose oxidize method, and HbA_1_c was measured by a high-pressure lipid chromatographic assay (VARIANT, BIO-RAD Lab., Hercules, CA, USA) that participated in the Chinese Ministry of Health Quality Assessment Program.

### Ophthalmic examination and classification of diabetic retinopathy

All participants who underwent OGTTs received eye examinations by an ophthalmologist and had a bilateral retinal photograph taken of the fundus through dilated pupils. Two 45° color digital images of the retina were taken of each eye by a technologist using a Topcon TRC-NW7SF fundus camera (Topcon, Tokyo, Japan) ophthalmic digital imaging system. The first image was centered on the macula, and the second was centered on the optic nerve. The photographs were graded by the two qualified ophthalmologists of Eye Center of Capital Medical University, Beijing Tongren Hospital according to the international clinical diabetic retinopathy severity scale [11]: (i) no retinopathic changes; (ii) mild non-proliferative retinopathy (NPDR); (iii) moderate NPDR; (iv) severe NPDR; and (v) proliferative retinopathy (PDR). The degree of diabetic retinopathy was determined according to the grading in the worse eye. The ophthalmologists grading the photographs were blinded to subjects' glucose and HbA_1_c levels.

### Statistical analysis

We used two different methods to estimate glycemic cut-off point associated with DR: Joinpoint regression and maximizing the sensitivity and specificity. Deciles, a widely used approach, were used to categorize FPG, 2-h PG and HbA_1_c and the prevalence of retinopathy for each subset was calculated. Joinpoint regression, in which the relationship between the dependent and independent variables is modeled as piecewise linear phases, is used to estimate changes in trend data [12]. Logistic regression was applied to test the relationship between glycemia and DR. To compare the ability of FPG, 2-h PG and HbA_1_c measurements to detect the presence or absence of retinopathy over a range of values, we calculated receiver operating characteristic (ROC) curves and compared the areas beneath them. ROC curves were also used to calculate the sensitivity, specificity of different measurements. A glycemic cutoff point's “sensitivity” was defined as its ability to correctly identify subjects with DR, while the “specificity” of a glycemic cutoff point was defined as its ability to correctly identify subjects who do not have DR. We also calculated glycemic cutoff levels defined by maximizing the sensitivity and specificity to identify diabetic retinopathy compared with other studies.

All statistical analyses were conducted with the software package SPSS version 11.5 (SPSS Inc., Chicago, IL, USA) for Windows, MedCalc version 11.4 (http://www.medcalc.be) and ljr package of R software (http://www.r-project.org). A two-sided p value of less than 0.05 was considered statistically significant.

## Results

Table 1 shows the characteristics of all study cohorts. Patients with DR rated significantly higher in age, BMI, FPG, 2-h PG, HbA_1_c and blood pressure compared with subjects without DR. Of the 2551 study participants, 74 (2.90%) subjects were found to have diabetic retinopathy. Mild NPDR, moderate NPDR, severe NPDR and PDR were found in 37 (1.45%), 28 (1.10%), 2 (0.08%) and 7 (0.27%) subjects respectively. Of the total of 3760 subjects, 2592 subjects (68.9%) consented to participate in the study. We compared the age, gender and FPG between the subjects who did participate in the study and those who did not and no significant differences in these parameters were observed.

**Table 1:** Characteristics of analytic population by diabetic retinopathy status.

	No retinopathy	Retinopathy	P
N	2477	74	
Age (year)	48.62±12.11	54.60±8.53	<0.001
Men (%)	48.77	47.30	>0.05
FPG (mmol/l)	6.56±1.70	10.50±3.72	<0.001
2-h PG (mmol/l)	8.13±4.51	18.14±7.35	<0.001
HbA_1_c (%)	6.00±1.07	8.67±2.22	<0.001
SBP (mmHg)	139.00±20.30	152.36±22.61	<0.001
DBP (mmHg)	85.06±11.04	88.04±11.97	<0.05
BMI (kg/m^2^)	25.85±3.69	26.27±3.03	>0.05
Hypentension (%)	53.65	75.68	<0.001

Values are means (standard deviation) or n (%).

Comparison between any two groups by unpaired t-test or chi-square test:

Abbreviations: FPG: fasting plasma glucose; 2-h PG: 2-h post oral glucose load plasma glucose; SBP: Systolic pressure; DBP: Diastolic pressure; BMI: body mass index.

**Figure 1:**
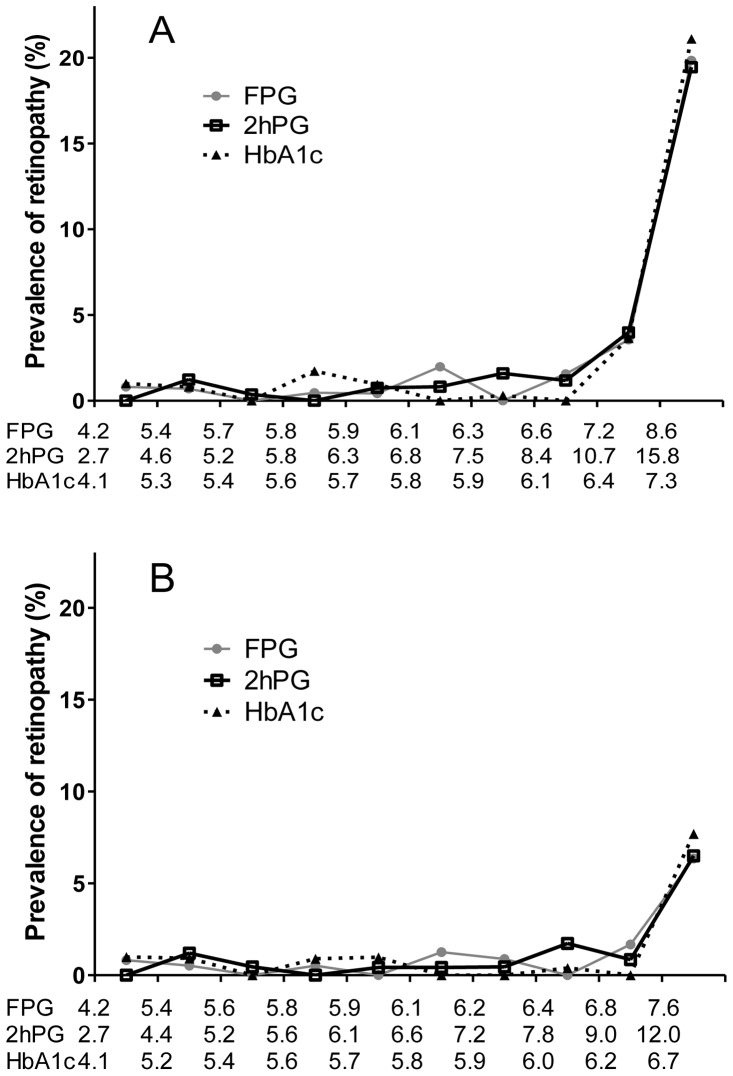
Prevalence of retinopathy by deciles of the distribution of FPG, 2-h PG and HbA_1_c levels for the total sample population (A) and the subpopulation excluding individuals receiving antihyperglycemic medication (B). The x axis labels indicate the lower limit of each decile group.

Figure 1 shows the prevalence of DR by deciles of the distribution of the FPG, 2-h PG and HbA_1_c levels. All three measures of glycemia were strongly associated with retinopathy, and the prevalence increased significantly between the eighth and the ninth decile of each variable (p<0.001), corresponding to an FPG of ≥7.2 mmol/l, a 2-h PG of ≥10.7 mmol/l, and HbA_1_c levels of ≥6.4%. The prevalences of DR for FPG, 2-h PG, HbA_1_c of DR in the ninth decile were 3.56%, 3.98% and 3.64% respectively, while those in the eighth decile were 1.56%, 1.18% and 0 respectively. Logistic regression models adjusted for sex, age, BMI, and hypertension status confirmed a statistically significant difference for DR compared with those below the cutoff points for FPG (OR 15.6 [95% CI: 8.7–27.9]; p<0.001), for 2-h PG (OR 16.2 [95% CI: 8.8–29.6]; p<0.001), and for HbA_1_c (OR 25.2 [95% CI: 12.8–49.6]; p<0.001). After excluding 230 individuals who were receiving antihyperglycemic medication, the prevalence of DR increased significantly between the eighth and the ninth decile of FPG (p<0.001); whereas the prevalence of DR increased significantly between the ninth and the tenth decile of 2-h PG and HbA_1_c (p<0.001), corresponding to an FPG of ≥6.8 mmol/l, a 2-h PG of ≥12.0 mmol/l, and HbA_1_c levels of ≥6.7%.

**Figure 2:**
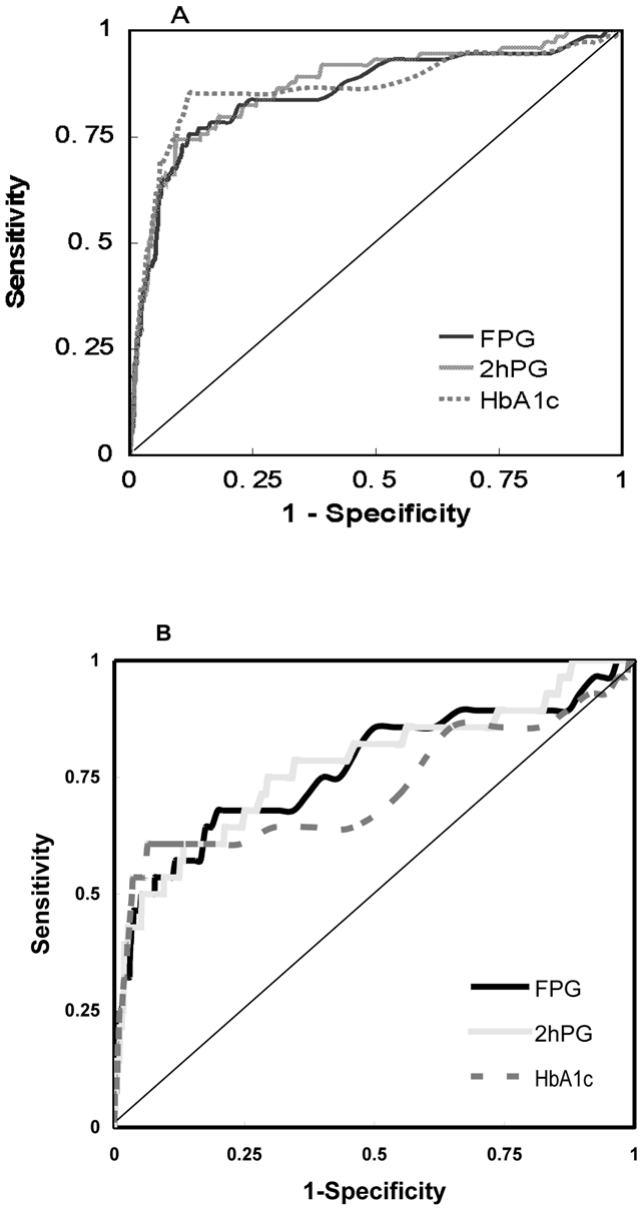
Receiver operating characteristics (ROC) curves for FPG, 2-h PG and HbA_1_c measures for detecting the presence of diabetic retinopathy in total sample population (A) and the subpopulation excluding individuals receiving antihyperglycemic medication (B).

Figure 2 shows the ROC curves for FPG, 2-h PG and HbA_1_c in detecting DR. The area under the ROC curve for 2-h PG was 86.9% (95% CI: 82.2–91.7) and was not significantly larger than that for FPG (85.4%; 95% CI: 80.0–90.7; p = 0.501) and that for HbA_1_c (86.4%; 95% CI: 80.8–92.0; p = 0.796). After excluding individuals receiving antihyperglycemic medication, the area under the ROC curve for 2-h PG was 77.6% (95% CI: 67.0–88.1), for FPG was 76.8% (95% CI: 65.8–87.8), for HbA_1_c was 72.5% (95% CI: 59.7–85.2). All three measures of glycemia also showed no significant differences for detecting DR (p = 0.891 for FPG and 2-h PG, p = 0.341 for 2-h PG and HbA_1_c, p = 0.281 for FPG and HbA_1_c).

Table 2 shows the optimal cutoff points of these three measures for identifying DR using Joinpoint regression, maximizing the sensitivity and specificity, and WHO criteria. In the total sample population, the sensitivities and the specificities of these cutoff points of FPG and 2-h PG using Joinpoint regression for our study were similar with WHO criteria. The sensitivity and specificity for the HbA_1_c level of 6.4% was slightly higher than that for the FPG and 2-h PG. The cutoff points for the three measures by maximizing the sensitivity and specificity were higher than by Joinpoint regression, and they had high specificities but low sensitivities. After excluding 230 individuals who were receiving antihyperglycemic medication, only 28 patients with DR were left. The sensitivities of these three cutoff points were very low regardless of what methods were used in this subpopulation. In order to see if the glucose cutoff points associated with DR may be affected by level of glucose control in the study population, we reanalyzed data excluding individuals whose glucose control were very poor. After excluding individuals whose HbA_1_c exceeded 8.5% (124 subjects),we found that glycemic cutoff levels defined by maximizing the sensitivity and specificity changed to 7.5 mmol/l, 9.2 mmol/l and 6.8% for FPG, 2-h PG and HbA_1_c, respectively.

**Table 2:** Performance of various cutoff points in detecting retinopathy in both the total sample population (left) and the subpopulation of those not receiving antihyperglycemic medication (right).

	Total sample population			Subpopulation not receiving antihyperglycemic medication		
Joinpoint regression	FPG (mmol/l)	2-hPG (mmol/l)	HbA_1_c(%)	FPG (mmol/l)	2-hPG (mmol/l)	HbA_1_c(%)
Cutoff point	7.2	10.7	6.4	6.8	12.0	6.7
Sensitivity	0.784	0.784	0.851	0.643	0.536	0.607
Specificity	0.830	0.821	0.821	0.814	0.905	0.916

Maximum sensitivity plus specificity						
Cutoff point	7.8	15.0	6.8	6.7	10.6	6.9
Sensitivity	0.757	0.743	0.851	0.678	0.607	0.607
Specificity	0.879	0.906	0.880	0.801	0.867	0.936

WHO criterion						
Cutoff point	7.0	11.1		7.0	11.1	
Sensitivity	0.784	0.770		0.571	0.536	
Specificity	0.807	0.833		0.853	0.882	

Abbreviations: FPG: fasting plasma glucose; 2-h PG: 2-h post oral glucose load plasma glucose.

## Discussion

The current guidelines for diabetes diagnosis were determined by examining the relationship between glycemia and DR. Cutoff points were calibrated from the results of several population-based studies [1], [3], [5]. However, no information has yet been made available for the Chinese population. Using representative population-based data, we examined the associations of FPG, 2-h PG and HbA_1_c with DR prevalence in the Chinese population. For all measures of glycemia, we identified points at which retinopathy prevalence began to rise sharply. Our results showed a dramatic increase in the prevalence of DR between the eighth and ninth deciles of each variable (FPG, 2-h PG and HbA_1_c). Retinopathy prevalence increased precipitously when FPG exceeded 7.2 mmol/l, when 2-h PG exceeded 10.7 mmol/l, and when HbA_1_c exceeded 6.4%. We also calculated glycemic cutoff levels defined by maximizing the sensitivity and specificity. However, we found that this way of analysis favored specificity, and the sensitivity was low. The conclusions of Joinpoint regression had balanced sensitivity and specificity. In addition, the cutoff levels of FPG and 2-h PG of Joinpoint regression were approximately consistent with the current WHO criteria. Therefore, we recommend glycemic cutoff levels derived by Joinpoint regression as the suitable measure in DR risk evaluation for the Chinese population.

Optimal plasma glucose thresholds for diabetes diagnosis can vary between populations. FPG cutoff levels of 6.8 mmol/l and 6.7 mmol/l equivalent to the 2-h PG criterion of 11.1 mmol/l were found in studies of Pima Indians [5] and NHANES III participants [3], respectively. Another study found that an HbA_1_c threshold of 6.3% may be acceptable as a diagnostic criterion for diabetes in the Chinese population [13]. However, a limitation of that study was that it did not use the presence of DR as the true diabetes state (gold standard). The only study [14] in an East Asian population using ophthalmic examination showed that the optimal cutoff levels for diagnosis of diabetes were 6.4 mmol/l for FPG, 11.1 mmol/l for 2-h PG, and 5.7% for HbA_1_c according to maximizing the sensitivity and specificity in a Japanese population. There are some reasons for the differences in these findings. Firstly, the sensitivity and tolerability to glucose load has been shown to vary between populations [6], [7], [8]. Furthermore, glycemic cutoff points associated with DR may also be affected by the level of understanding and disease control in the study population. In our study population, subjects with retinopathy had very poor glucose control (10.50 mmol/l for FPG, 18.14 mmol/l for 2-h PG and 8.67% for HbA_1_c). This situation may also influence the glycemic cutoff levels. After excluding individuals whose HbA_1_c exceeded 8.5%, we found that glycemic cutoff levels defined by maximizing the sensitivity and specificity decreased from 7.8 to 7.5 mmol/l by FPG and from 15.0 to 9.2 mmol/l by 2-h PG. Therefore, this is one of the important reasons why our glycemic cutoff levels were higher than those in the Japanese study. It also demonstrates that we should pay more attention to glucose control in Chinese diabetes patients.

Existing diagnostic methods of diabetes recommended for us in clinical practice include FPG, 2-h PG, and HbA_1_c. However, which of these three should be the preferred method remains a debateable topic. In the Egyptian study, FPG and 2-h PG both showed stronger associations with DR than did HbA_1_c [1]. In contrast, in the Pima Indian study and Japanese study, ROC analysis showed that the area under the curve for 2-h PG was slightly but not significantly larger than that for FPG and that for HbA_1_c [5], [14]. In line with the diagnostic method recommended by the American Diabetes Association and an International Expert Committee [15], our findings suggest that all three measures are effective for diagnostic purposes, and that the FPG or HbA_1_c alone are acceptable alternatives to 2-h PG, which is complicated to measure by OGTT. The OGTT has been the preferred test for diagnosing diabetes in epidemiological studies for over 50 years [5]. This choice has persisted despite the inconvenience of the test. Our findings suggest that HbA_1_c or FPG concentration could be preferable glycemic tests for diagnostic purposes. Additionally, HbA_1_c or FPG are easier to obtain than 2-h PG. Although the prevalence of diabetes has dramatically increased in recent years in China, the disease remains underdiagnosed [9], [10]. More efficient identification of people with diabetes is thus essential to allow timely provision of treatment. FPG is a suitable choice to diagnose diabetes especially in rural China because of its convenience and inexpensiveness. HbA_1_c is more convenient because it can be done at any time without fasting or other preparation of the patient, which makes diagnosis on the same day possible [13]. Although FPG and HbA_1c_ identify different individuals, our study has demonstrated that FPG and HbA_1c_ tests are equally reliable methods to detect persons with a high-risk of DR. Therefore, we recommend using FPG or HbA_1_c for the diagnosis of diabetes in the Chinese population as a more convenient alternative to OGTT.

There are several potential limitations in this study. Firstly, it is a cross-sectional study. The relationship between the onset of DR and the three measures of glycemia in this study is yet to be elucidated. Secondly, our results could be biased by the participation rate of 68.9%. However, no significant differences of age, gender and FPG were observed between the subjects who did participate in the study and those who did not. Finally, some previous studies have included those who were receiving medication [14], [16], [17], and others have excluded them [5], [18]. Excluding those receiving treatment eliminates the treatment effect, but can change the characteristics of the population with diabetes [1]. We performed analyses with and without inclusion of individuals who were receiving antihyperglycemic medication respectively. However, after excluding individuals receiving antihyperglycemic medication, the sensitivities of cutoff points of glycemia were very low due to the relatively low number of DR subjects not on antihyperglycemic treatment.

In conclusion, our population-based study examined the association of the three glycemic measures with retinopathy and provided new information on defining cutoff points for diagnosing diabetes in the Chinese population. Our results suggest that considerations of the risk of microvascular complications (DR), FPG concentration or HbA_1_c concentration are equally reliable methods as measuring 2-h PG for the purposes of diagnosing diabetes in the Chinese population.
